# The Early Psychological Impacts of the Deepwater Horizon Oil Spill on Florida and Alabama Communities

**DOI:** 10.1289/ehp.1002915

**Published:** 2011-02-17

**Authors:** Lynn M. Grattan, Sparkle Roberts, William T. Mahan, Patrick K. McLaughlin, W. Steven Otwell, J. Glenn Morris

**Affiliations:** 1 Department of Neurology, School of Medicine, University of Maryland, Baltimore, Maryland, USA; 2 Florida Sea Grant Extension Program, Institute of Food and Agricultural Sciences, University of Florida, Gainesville, Florida, USA; 3 Department of Medicine, College of Medicine and; 4 Emerging Pathogens Institute, University of Florida, Gainesville, Florida, USA

**Keywords:** disasters, environmental epidemiology, occupational health, petroleum products, risk perception

## Abstract

**Background:**

Although public concern has focused on the environmental impact of the Deepwater Horizon oil spill, the public health impact on a broad range of coastal communities is minimally known.

**Objective:**

We sought to determine the acute level of distress (depression, anxiety), mechanisms of adjustment (coping, resilience), and perceived risk in a community indirectly impacted by the oil spill and to identify the extent to which economic loss may explain these factors.

**Methods:**

Using a community-based participatory model, we performed standardized assessments of psychological distress (mood, anxiety), coping, resilience, neurocognition, and perceived risk on residents of fishing communities who were indirectly impacted (*n =* 71, Franklin County, Florida) or directly exposed (*n =* 23, Baldwin County, Alabama) to coastal oil. We also compared findings for participants who reported income stability (*n =* 47) versus spill-related income loss (*n =* 47).

**Results:**

We found no significant differences between community groups in terms of psychological distress, adjustment, neurocognition, or environmental worry. Residents of both communities displayed clinically significant depression and anxiety. Relative to those with stable incomes, participants with spill-related income loss had significantly worse scores on tension/anxiety, depression, fatigue, confusion, and total mood disturbance scales; had higher rates of depression; were less resilient; and were more likely to use behavioral disengagement as a coping strategy.

**Conclusions:**

Current estimates of human health impacts associated with the oil spill may underestimate the psychological impact in Gulf Coast communities that did not experience direct exposure to oil. Income loss after the spill may have a greater psychological health impact than the presence of oil on the immediately adjacent shoreline.

The Deepwater Horizon oil platform explosion and spill on 20 April 2010 generated substantial concerns about the ecological impact on the U.S. Gulf Coast environment. For 5 months, almost 5 million barrels of oil spilled into the Gulf of Mexico, reaching > 600 miles of the Gulf Coast shoreline in Florida, Alabama, Louisiana, and Texas (Devi 2010; McCauley 2010; Schmidt 2010). It was the largest offshore spill in U.S. history (McCauley 2010). The oil spill disrupted the region’s fishing industry, destroyed renewable natural resources, and caused significant mortality of fish and wildlife. Numerous questions were also raised about the potential impact of the spill on human health in oil-exposed regions and surrounding communities.

Using a community-based participatory research model, our investigators worked with community agencies and leaders from two Gulf Coast fishing communities (Franklin County, Florida and Baldwin County, Alabama) to develop and implement a formal investigation of the acute psychological distress, neuropsychological baseline status, and personal resources for adjustment and adaptation of local residents. Extant data suggest that after disasters, mental health problems are most likely to appear after the acute crisis has abated (see Rubonis and Bickman 1991 for review; van den Berg et al. 2005). However, real-time acute psychological data are rarely available. These data are particularly important, as the psychological impacts of an oil spill can be as substantive as the ecological impacts (Arata et al. 2000; Gill and Picou 1998; Palinkas et al. 1992, 1993; Sabucedo et al. 2009).

Because oil never reached Franklin County shores, effects of the disaster would have been indirect (i.e., not due to direct exposure to the oil) but may have been significant nonetheless. Residents observed daily media reports about the spill, provided clean-up assistance in other Gulf communities, and actively engaged in protective environmental activities in anticipation of oil reaching their shores. Fears about seafood safety led to a dramatic reduction in local seafood harvesting, forcing layoffs in packing houses and transportation because of a lack of product.

The potential for significant psychological sequelae after indirect exposure to oil spills and other environmental disasters has been well documented. These parallel the psychological distress associated with direct disaster exposure and include symptoms of depression, anxiety, and post-traumatic stress disorder (PTSD) (Baschnagel et al. 2009; Carballo et al. 2006; Chung et al. 2005; Dixon et al. 1993; Gallacher et al. 2007). Three psychological factors consistently emerge as possible mediators of psychological distress after oil spills or disasters. These include coping, or the process through which people regulate distress and manage the problems related to it (Benight et al. 1999; Chung et al. 2005); resilience, the ability to bounce back after crisis (Bonanno et al. 2006; Rajkumar et al. 2008); and perceived risk, the way people approach, think about, and interpret the risks in their environment (Gallacher et al. 2007; Moffatt et al. 2000; Renn 2004). These processes guide the way an individual views the risks and challenges of the situation, define their predisposition to maintain emotional stability in the midst of crisis, and provide the basic tools for problem solving, planning, and adaptation.

The most severe, lasting, and pervasive psychological effects are often found after disasters that engender serious and ongoing financial problems (Nandi et al. 2009; Norris et al. 2002). Economic resource loss has been associated specifically with long-term psychological and mental health symptoms after both the Exxon Valdez and Prestige oil spills (Arata et al. 2000; Sabucedo et al. 2009). We hypothesized that income loss during the Deepwater Horizon oil spill disaster would be associated with similar acute psychological reactions.

Many factors impacting psychological reactivity after oil spills are potentially modifiable. With this in mind, our community–academic partnership was initiated to identify people at greatest risk for mental health problems for early public health intervention. The study objective was 2-fold: *a*) to determine the acute level of psychological distress (depression, anxiety), mechanisms of adjustment (coping, resilience), and perceived risk of individuals in a community who were indirectly impacted by the Deepwater Horizon oil spill disaster; and *b*) to determine whether participants who sustained economic loss as a result of the oil spill had greater evidence of psychological distress, reduced capacity for adjustment (coping, resilience), and greater perceived risk than persons who were economically stable. We hypothesized that *a*) in Gulf coastal communities, the psychological distress (depression, anxiety), mechanisms of adjustment (coping, resilience), and perceived risk (environmental worry) associated with indirect impact would be similar to that of direct exposure to the oil spill disaster; and that *b*) people with oil spill–related economic losses would have more psychological distress, have less resilience, be more likely to use maladaptive coping strategies, and report more risk concerns than those with economic stability during the oil spill crisis.

## Methods

This study was undertaken as part of a larger ongoing effort being conducted by the University of Florida to assess the acute environmental and health impacts of the spill among persons living in fishing communities along the Florida and adjacent Alabama coast. Using a community-based participatory model, we developed and implemented the project in collaboration with local community and religious leaders, mental health coalitions, trade associations, and the University of Florida, Franklin County extension service. Our community partners also provided insight into measurement selection and adaptation, the interpretation of our findings, and recommendations for outreach and intervention.

### Participants and procedures

Study participants in Franklin County, Florida, included 71 adult volunteers with permanent residence in the county [population, 11,280; towns of Apalachicola, Eastpoint, and Carrabelle, on Apalachicola Bay; see map in Supplemental Material, Figure (doi:10.1289/ehp.1002915)] who sustained an indirect impact or exposure to the oil spill. Indirect impact was defined as living in a community where oil did not reach the coastline but significantly impacted fishing and recreation/tourism economies and required reallocation of resources to protect their shellfish beds, wildlife, and other coastal resources. Recruitment was targeted toward adults (18–75 years of age) working in the fishing/seafood and tourism/service industries, as well as family members, recreational fishers or harvesters, and retirees who lived and recreated in the community. Persons were excluded if they had a neurologic or psychiatric condition that would preclude understanding the informed consent or examination procedures. Recruitment was through advertisement on the local radio station (Oyster Radio) and contacts with members of the local fishing industry though the University of Florida Extension Office.

The direct exposure comparison group (persons living or working in a community where spilled oil reached the shoreline) included 23 participants from Baldwin County, Alabama (towns of Bon Secour and Foley on Bon Secour Bay/Mobile Bay; population, 10,059). Our community partner in Bon Secour was the local office of the Alabama Seafood Association. Members (and families) of the association were contacted by telephone (by the local association secretary) and invited to participate in the study. Exclusion criteria were the same as the primary study group.

All study participants underwent all study procedures. The community partners identified exam locations that included three sites for the primary, indirect exposure study group (two churches and the Carrabelle City Town Hall) and one church for our comparison group. A team of three examiners, formally trained in psychological and neuropsychological assessment, administered the standardized cognitive and psychological interviews and procedures. The team included a licensed psychologist, who supervised two additional research assistants responsible for reading forms and paper-and-pencil measures to participants with literacy or vision difficulties. Written informed consent was obtained from all participants in compliance with all applicable U.S. requirements according to standard procedures required by the University of Maryland and the University of Florida institutional review boards. The examination procedures took approximately 90 min and included a standard interview and formal neuropsychological, psychosocial, and risk perception measures. All measures were selected based on *a*) previously established reliability and validity for the constructs they measure and the populations to which they were applied; *b*) ease of administration in the field; *c*) repeatability for prospective studies; and *d*) ability to assess the construct of interest with minimal participant burden. Participants were reimbursed $40.00 for study participation. We performed data analyses using the PASW Statistics Package 18 (IBM, Chicago, IL); an alpha level of 0.05 was established as the cutoff for statistical significance.

### Demographic, medical, and psychosocial history

Basic demographic, occupational, medical, psychiatric, and drug/alcohol history data were collected using a modified Boston Occupational and Environmental Neurology Questionnaire (BOENQ) (Feldman 1999) and Brief Michigan Alcohol Screening Test (BMAST) (Pokorny et al. 1972). The BOENQ was modified to include questions relevant to fishing occupations and income since the oil spill. The BMAST was modified to include questions about drug use and postspill alcohol consumption. Psychiatric history was determined by self-reported lifetime history of treatment for depression and/or anxiety through therapy, hospitalization, or medication.

### Economic loss

Economic loss was determined for each participant based on their responses to the following two questions: Have you lost any income since the oil spill? (BOENQ, dichotomous response choice, with follow-up for reason attributed to reduced income). What has been the biggest impact of the oil spill? (Health and Coastal Environment Questionnaire-V, open-ended question). Participants were assigned to the economic loss group if they indicated they lost income since the oil spill, the income loss was related to the oil spill, and the biggest impact of the oil spill on their life was economic.

### Neuropsychological battery

The neuropsychological battery evaluated neurocognitive functions within the context of possible exposure to oil and chemical dispersants. Cognitive impairments or associated exposures, if they exist, can potentially confound the assessment and interpretation of the psychological and behavioral variables of interest. The neuropsychological screening battery consisted of tests from the World Health Organization Neurobehavioral Core Test Battery, which has been recommended for use in studies of neurotoxin exposures (Johnson 1987). This test included the Lafayette Pegboard (Lafayette Instrument Company 2002) to assess psychomotor speed and dexterity; Digit Span (Wechsler Adult Intelligence Scale–3rd edition; WAIS-3) (Wechsler 1997) to measure simple attention; Symbol Digit Modalities Test (Smith 1982) to measure clerical speed and accuracy; Stroop Color Word Test (Golden and Freshwater 2002) to determine response inhibition; and the Trailmaking Test (Reitan 1992) to assess divided attention and mental flexibility. Age, sex, and education corrections were applied in scoring.

### Psychological distress

We used the Profile of Mood States (POMS) (McNair et al. 1992) to assess transient, fluctuating mood states and to determine current mood state (including anxiety and depression) in our study groups. Administration procedures require the respondent to read a list of 60 words (e.g., “friendly,” “tense,” “helpless”) or short phrases (e.g., “unable to concentrate,” “uncertain about things”) that describe feelings that people have and then indicate on a five-point Likert-type scale whether they experienced each feeling or state “since the oil spill, including today (0 = not at all, 1 = a little, 2 = moderately, 3 = quite a bit, 4 = extremely).” Responses were summed for six scales (tension/anxiety, depression, anger, vigor, fatigue, confusion) and total mood disturbance. Standard procedure for scoring this measure involves converting raw scores to *t*-scores (mean ± SD, 50 ± 10) referencing an adult, normative database provided in the manual (McNair and Heuchert 2005). With the exception of vigor, higher scores indicate more adverse outcome on the subscale. Because clinical interpretation was of interest to our community partners, standard cutoffs for the POMS were applied (SD, 1.5) (Nyenhuis et al. 1999) to identify persons with suspected psychopathology or needing special attention. POMS protocols were reviewed after each administration. Persons who reported multiple symptoms related to depression or anxiety received a follow-up clinical interview by a licensed psychologist to determine if they were in acute distress or required immediate intervention and/or referral. The POMS has been widely used to evaluate mood state in a variety of normal, psychiatric, medical, and disaster- related neurotoxicology populations (Bowler et al. 1994a, 1994b; Bowler et al. 1998; McNair and Heuchert 2005). It is sensitive to mood change and has excellent utility in studies where repeated measures are anticipated.

### Coping style

Coping strategies are used to describe the way people respond to stress. In this investigation, we studied the coping strategies people used during the oil spill. Coping was assessed using the Brief COPE questionnaire (Carver 1997). The questionnaire comprised 28 items such as “I’ve been concentrating my efforts on doing something about the situation,” “I’ve been using alcohol or other drugs to make myself feel better,” and “I’ve been praying or meditating.” Participants were asked to indicate how often they used each strategy to cope since the oil spill on a four-point Likert-type scale ranging from 1 (“I haven’t been doing this at all”) to 4 (“I have been doing this a lot”). For data analysis, the sum of the items were clustered into 14 coping strategies: self-distraction, active coping, denial, substance use, emotional support seeking, instrumental support seeking, behavioral disengagement, venting, positive reframing, planning, humor, acceptance, religion, and self-blame. Scores for each strategy may range from 1 to 8, and a higher score indicates a greater use of the coping strategy. The Brief COPE was validated on a sample of adults participating in a study of psychological recovery after Hurricane Andrew (Carver 1997). The psychometric properties of the Brief COPE and its precursor, the COPE (Carver et al. 1989) have been well established in both normal and clinical populations. The 14 coping scales are intended to be interpreted independently in relation to variables under study (Carver 1997).

### Resilience

***“***Resilience” refers to the ability to bounce back from adversity; for the purpose of this study, “resilience” is operationally defined by responses on the Connor-Davidson Resilience Scale (CD-RISC, short form) (Campbell-Sills and Stein 2007). The CD-RISC requires participants to consider 10 statements believed to be characteristic of a resilient person and rate them on a 0–4 scale based on how closely the statement resembles their current state. Item examples include “I can deal with whatever comes,” “I tend to bounce back after illness or hardship,” and “I can stay focused under pressure.” This measure is scored by summing the responses. The total score range is 0–40, with the higher score reflecting greater resilience. The CD-RISC, short form and its predecessor scale were validated on a community sample, psychiatric outpatients, clinical trials for the treatment of PTSD, and victims of childhood trauma (Connor 2006). The measure has sound psychometric properties and distinguishes between people with greater and less resilience (Campbell-Sills and Stein 2007; Connor and Davidson 2003).

### Perceived risk

Several questions or questionnaires have been developed and used to measure perceived risk or aspects of it (e.g., environmental worry). For the most part, these measures tend to be study specific. None have been widely used, gained general acceptance, or established primacy in the field. In the present study we used the Health and Coastal Environment Questionnaire-V (HCEQ-V) (Roberts et al. 2007), which was previously developed and validated in several coastal communities facing threats of marine-based toxins. It assesses three facets of risk perception: environmental worry, environmental safety, and environmental knowledge. It also identifies community sources of trusted information. The HCEQ-V is a structured 19-item survey that may be adapted to specific coastal environment threats. It is composed of forced choice (“Scientists will succeed in providing ways to restore the natural environment,” response: yes, no, don’t know”) and open-ended questions (“What is the biggest problem(s) you have related to the oil spill?”). The question regarding who the respondent turns to for reliable heath information allows for multiple responses. The most frequently selected items are reported here. The survey was field tested and modified for content and language based on community feedback [see the HCEQ-V questionnaire in Supplemental Material (doi:10.1289/ehp.1002915)].

## Results

### Recruitment

Ten percent of the persons contacted for participation in the indirect impact group (Franklin County) declined participation for the following reasons: They were out of town (1%), busy managing oil spill–related problems during the period of our evaluations (4%), or worried that our research was funded by BP (5%). In the direct exposure group (Baldwin County/Bon Secour), approximately 12% of the people contacted declined participation. The reasons stated were involvement in oil spill cleanup operations (5%), managing other oil spill–related problems during the evaluation period (2%), or worried that participation would represent a violation of contractual confidentiality agreements with BP (5%).

### Demographic and background information

Table 1 contains the demographic and basic descriptive information for study participants by key variables: exposure community (indirect, direct) and income status (income stable, income loss). There was a significant difference between age, education, and occupation between the two community exposure groups. Twenty-two of the 23 participants in the direct exposure group were men compared with only half of those in the indirect exposure group, and most were professional fishermen. The indirect exposure group included more retired professionals and persons in service/tourism industry, was older, and had a higher average educational level than the direct exposure group. Only one participant in the indirect exposure group was involved in spill cleanup activity compared with 70% of those in the direct exposure group. Economic loss was reported by 55% and 35% of those in the indirect and direct exposure groups, respectively (*p* = 0.09). We found no significant differences between the economic resource groups on any of the demographic or background measures. Base rates for lifetime history of treatment for depression, anxiety, or current alcohol problems were similar for all groups.

### Neuropsychological test scores

We observed no statistically significant differences between the exposure groups with respect to simple attention (Digit Span), response inhibition (Stroop), clerical speed and accuracy (Symbol Digit Modalities Test), or divided attention and mental flexibility (Trails A and B) [see Supplemental Material, Table 1 (doi:10.1289/ehp.1002915)]. The direct oil exposure group was slower on the pegboard task (psychomotor speed and accuracy) than the indirect exposure group, but the differences were not clinically significant. Cognitive scores were comparable between the income groups, and cognitive performance scores for all four groups were within the normal and expected ranges.

### Mood

There were no statistically significant differences in subscale scores between the exposure groups (Table 2). We observed significant differences between the income groups on the POMS tension/anxiety, depression, anger, fatigue, confusion, and total mood disturbance subscales. The income loss group consistently scored higher on these scales than those with stable incomes, suggesting more distress in multiple psychological domains. Collapsing across all groups, there were no clinically relevant differences between participants with a lifetime history of depression or anxiety (*n =* 9) and those who were never diagnosed or treated for anxiety or depression (*n =* 85). When standard cutoffs suggesting clinical impairment were applied, we found indicators of clinically significant anxiety and depression across all study groups (Table 2). A sizable proportion of participants in both community exposure groups (50% indirect impact, 35% direct exposure) had scores suggestive of clinically significant depression. Although the rate of clinical impairment was not statistically different between the exposure groups (χ^2^ *=* 1.59, *p =* 0.21), the depression rates of both groups were higher than reported in 2008 in the region: 9.8% Florida and 13% Alabama (Centers for Disease Control and Prevention 2010).

The number of persons with clinically significant depression was elevated in both income groups (30% income stable, 62% income loss) relative to regional base rates (Centers for Disease Control and Prevention 2010), with significantly more people in the income loss group meeting the criteria for probable depression (Table 2). With respect to tension/anxiety, 24% of people in the income stable group and 65% of people in the income loss group had clinically significant scores (*p* < 0.001).

### Mechanisms of adjustment

Active coping was used significantly more by the direct exposure group than by the indirectly impacted group (Table 2). Participants in the direct exposure group were more likely to agree with the following statements: “I’ve been concentrating my efforts on doing something about the situation I’m in” and “I’ve been taking action to try to make the situation better.” When the income groups were compared, those with income loss were significantly more likely to use behavioral disengagement as a coping strategy. Behavioral disengagement involves giving up trying to deal with or cope with the problem (Carver 1997).

We found no difference in the resilience scores between exposure groups. However, the income loss group had a significantly lower mean resilience score than the income stable group. Finally, when baseline history of anxiety or depression was considered across all groups, there was no statistically significant difference in resilience scores between participants with (*n =* 9, mean resilience = 27) or without (*n =* 85, mean resilience = 30) a prior history of anxiety or depression (*p* = 0.10).

### Perceived risk

Results of the perceived risk survey (Table 3) indicate that 98–100% of study participants as a whole worried about the impacts of the oil spill on the environment, seafood safety, and human health. A greater proportion of people in the indirectly impacted group, compared with the direct exposure group, thought BP would be successful in oil spill cleanup. Nine percent of the participants from the directly exposed community believed they were sickened by exposure to oil or dispersants, and 17% were uncertain or did not know. There was a significant difference between the exposure groups with respect to future health impacts. Of the people who believed there would be health impacts, 21% of participants in the indirectly exposed group believed the health effects would be only short term. In contrast, 100% of people who believed there would be health impacts in the directly exposed community thought the health impacts would be both short and long term (*p* = 0.03).

When asked where they obtained their most reliable information about oil spill–related health matters, > 75% of all participants indicated newspapers or television [see Supplemental Material, Table 2 (doi:10.1289/ehp.1002915)]. The department of health was viewed as a more reliable source of information by the indirectly impacted (25%) and income loss groups (32%) than by their comparison groups (4% and 9%, respectively). Local fishermen were considered a reliable source of information by the persons who sustained income loss (66% vs. 13%, *p* = 0.03). Finally, 22% of the direct exposure group considered BP to be a reliable source of information, compared with only 5% of the indirect exposure group (*p* = 0.01).

## Discussion

In this study we examined the effects of community oil exposure (direct, indirect) and income loss with respect to acute psychological distress, coping, resilience, and perceived risk after the Deepwater Horizon oil spill disaster. As hypothesized, during the spill, people living in a Gulf Coast community with indirect impact had elevated levels of anxiety and depression similar to those of people living in communities where oil reached their shores. When participants were divided by spill-related income loss, the expected differences in psychological distress emerged. People who suffered income losses as a result of the spill reported significantly more tension/anxiety, depression, anger, fatigue, confusion, and overall mood disturbance than their income-stable counterparts. The income loss group also had a higher rate of clinically elevated depression scores than any other study group. In summary, these data highlight the potentially profound psychological impact the Deepwater Horizon disaster had on coastal communities with indirect impact, particularly if they sustained economic loss.

Mechanisms of adjustment such as coping strategy and resilience are often viewed as buffers to the psychological impacts of stressful life events. In our study, the income loss group was more likely than the stable income group to use behavioral disengagement (giving up) as a coping strategy. Disengaging from coping efforts and other avoidant strategies has been associated previously with adverse psychological outcomes after disasters, including oil spills (Arata et al. 2000; Silver et al. 2002). The lowest resilience scores were also found in persons who sustained income loss and did not appear to be associated with baseline history of depression or anxiety. Resilience refers to the qualities that enable one to thrive despite adversity (Campbell-Sills and Stein 2007; Connor and Davidson 2003). It implies an inner strength thought to be protective against the development of psychiatric disorder (Rutter 1987). Income decline has been associated with reduced resilience and persistent psychological symptoms after disaster (Bonanno et al. 2007), thus suggesting that people with spill-related income loss might have fewer psychological resources for bouncing back.

Economic resource loss, socioeconomic adversity, and/or loss of job opportunities have been associated with course of depression, number of PTSD symptoms, or psychological distress after other disasters, including the Sierra Madre earthquake (California), Hurricane Hugo (South Carolina), and the 9/11 New York City terrorist attacks (Bonanno et al. 2006; Freedy et al. 1992, 1994; Kaniasty and Norris 1995; Nandi et al. 2009). Six years after the Exxon Valdez oil spill, Arata et al. (2000) examined economic resource loss within the context of a broader, conservation-of-resources stress model (Hobfoll 1989). They found resource loss (having to sell possessions) to be significantly correlated with anxiety, depression, and PTSD in commercial fishers after the Exxon Valdez spill (Arata et al. 2000). Socioeconomic factors such as income loss may have a profound impact on psychological adjustment and adaptation after oil spills.

With regard to perceived risk, both exposure groups and economic resource groups in the present study had similarly high levels of worry about the impact of the spill on the environment, human health, and seafood safety. Therefore, any direct relationship between environmental worry and acute psychological distress could not be examined. During the acute event, participants considered television and newspapers the most reliable source of human health information. People who sustained income loss were more likely to turn to the local fishermen and the department of health for their information. Meanwhile, BP was found to be a reliable source of information by the directly exposed community.

### Limitations

The primary limitations of this study are sample size and sampling procedures, which may have led to sampling biases. The indirect impact group was larger, older, better educated, and included more women than the comparison group (which focused on persons working directly on the water). The collective and acute nature of disaster creates a unique challenge for community-based public health research. During this real-time assessment of psychological distress, our community partners, who were also community leaders, were deeply entrenched in other disaster- related matters. This precluded the implementation of labor-intensive, systematic sampling procedures. Within the context of this research model, we used the best available recruitment methods to assemble the participant samples. The absence of preexposure data and the cross-sectional approach represent additional limitations that preclude the ability to directly establish a causal relationship between the oil spill and distress of community members.

### Implications

Impacts of oil spills extend beyond communities where oil reaches the shoreline. This underscores the need to extend public health education and outreach, psychological monitoring, and mental health services beyond the direct spill areas. From a mental health perspective, people at risk of income loss comprise a particularly vulnerable population. Therefore, this group should be specifically targeted for financial counseling and support, alternative employment opportunities, and psychological interventions. These interventions need to be immediately available in the communities where the impacted individuals live.

PTSD is the most often studied and most frequent and debilitating psychological disturbance that occurs after disasters (Galea et al. 2005). Longitudinal studies are needed to examine the range of factors associated with the development and/or persistence of PTSD or related disorders from the acute phase of psychological reactivity. Income loss, as well as other socioeconomic factors, should be considered in predictive models. Most people who are exposed to disasters do not develop PTSD or other chronic debilitating psychological conditions. Therefore, research on the factors associated with normal, adaptive recovery to disaster is also indicated. Subsequently, we could better identify target groups for varying levels of support or interventions. Finally, use of a community-based participatory model enabled the development of a sustainable community–academic relationship dedicated to improving the public health of participating oil spill communities.

## Figures and Tables

**Table 1:** Characteristics of study participants.

	Exposure			Income status		
Characteristic	Indirect (*n* = 71)	Direct (*n* = 23)	*p*-Value	Stable (*n* = 47)	Loss (*n* = 47)	*p*-Value
Sex (male)^a^	35 (49)	22 (96)	0.00	32 (68)	23 (53)	0.14
Age^b^	48.99 ± 16.45	41.91 ±11.16	0.02	49.32 ±16.92	45.19 ±13.97	0.20
Range	19–88	22–63		19–88	22–79	
Education^b^	12.39 ± 3.05	10.52 ± 1.86	0.01	12.26 ± 3.21	11.59 ± 2.56	0.27
Range	5–20	8–13		5–20	5–18	
Race^b^	NA	NA	0.31	NA	NA	0.40
Caucasian	64 (90)	22 (100)	NA	41 (89)	45 (96)	NA
African American	6 (9)	0 (0)	NA	4 (9)	2 (4)	NA
Native American	1 (1)	0 (0)	NA	1 (2)	0 (0)	NA
Occupation^a^	NA	NA	0.00	NA	NA	0.35
Fishing	26 (38)	20 (87)	NA	22 (47)	24 (53)	NA
Service/tourism	15 (22)	1 (4)	NA	7 (15)	9 (20)	NA
Retired	7 (10)	0 (0)	NA	5 (11)	2 (4)	NA
Relative of fish industry	2 (3)	0 (0)	NA	0 (0)	2 (4)	
Other	18 (27)	2 (9)	NA	12 (26)	8 (18)	NA
Economic loss^a^	39 (55)	8 (35)	0.09	NA	NA	NA
Spill cleanup participant^a^	1 (1)	16 (70)	0.00	9 (19)	8 (17)	0.79
Psychiatric history^a^						
Depression	5 (7)	1 (4)	0.65	1 (2)	5 (11)	0.09
Anxiety	4 (6)	2 (9)	0.60	4 (9)	2 (4)	0.40
Alcohol problem^a^,^c^	4 (10)	2 (9)	0.89	4 (13)	2 (4)	0.39

NA, not applicable. All characteristics are reported as frequencies (percentages) except for age and education, which are reported as mean ± SD.

a Probability associated with a chi-square test (two-tailed distribution).

b Probability associated with an independent samples *t*-test (two-tailed distribution).

c Potential alcohol problem was determined by using the criteria from the National Institute on Alcohol Abuse and Alcoholism.

**Table 2:** Psychosocial scores for study participants.

	Exposure			Income status		
Psychosocial measures	Indirect (*n* = 71)	Direct (*n* = 23)	*p*-Value^a^	Stable (*n* = 47)	Loss (*n* = 47)	*p*-Value^a^
POMS						
Tension/anxiety	56.89 ± 17.97	62.44 ± 11.33	0.17	53.23 ± 16.09	63.26 ± 15.94	0.00
Depression	55.70 ± 20.22	57.70 ± 12.99	0.66	51.90 ± 18.37	60.94 ± 18.14	0.02
Anger	56.13 ± 20.63	59.91 ± 13.24	0.41	53.17 ± 19.27	60.49 ± 18.30	0.05
Fatigue	49.41 ± 16.87	55.83 ± 12.95	0.10	47.43 ± 16.55	54.53 ± 15.15	0.03
Confusion	54.92 ± 20.16	60.78 ± 11.62	0.19	52.77 ± 17.93	59.94 ± 18.66	0.06
Vigor	40.44 ± 13.94	41.61 ± 10.16	0.71	40.74 ± 14.33	40.70 ± 11.83	0.99
Total mood disturbance	55.66 ± 20.07	61.13 ± 11.71	0.22	52.93 ± 18.12	61.06 ± 18.10	0.03

POMS suspected clinical impairment						
Tension/anxiety^b^,^c^	44	48	0.76	24	65	0.00
Depression^b^,^c^	50	35	0.21	30	62	0.00

Brief COPE						
Self-distraction	4.60 ± 2.10	4.40 ± 1.70	0.72	4.40 ± 2.10	4.60 ± 2.01	0.39
Active coping	4.70 ± 2.10	6.10 ± 2.00	0.01	5.40 ± 2.10	4.80 ± 2.20	0.27
Denial	4.00 ± 2.20	3.40 ± 2.00	0.25	3.40 ± 2.20	4.20 ± 2.20	0.09
Substance use	2.80 ± 1.40	2.70 ± 1.60	0.81	2.80 ± 1.40	2.70 ± 1.40	0.64
Use of emotional support	3.80 ± 1.70	3.60 ± 1.70	0.71	3.50 ± 1.60	4.00 ± 1.80	0.15
Use of instrumental support	3.80 ± 1.90	3.90 ± 1.80	0.82	3.70 ± 1.70	4.00 ± 2.00	0.40
Behavioral disengagement	3.20 ± 1.50	2.80 ± 1.40	0.28	2.70 ± 1.10	3.40 ± 1.70	0.02
Venting	4.00 ± 1.70	4.50 ± 1.60	0.18	3.90 ± 1.60	4.40 ± 1.70	0.17
Positive reframing	4.60 ± 1.90	5.10 ± 1.90	0.21	4.70 ± 2.00	4.70 ± 1.80	0.96
Planning	5.10 ± 1.90	5.80 ± 2.00	0.13	5.00 ± 1.90	5.60 ± 2.00	0.19
Humor	3.00 ± 1.50	4.00 ± 2.30	0.08	3.30 ± 1.70	3.20 ± 1.80	0.68
Acceptance	5.60 ± 1.90	6.30 ± 1.50	0.09	5.90 ± 1.80	5.70 ± 1.90	0.63
Religion	4.60 ± 2.30	5.60 ± 2.40	0.11	4.80 ± 2.40	5.00 ± 2.30	0.77
Self-blame	2.80 ± 1.30	2.80 ± 1.50	0.77	2.50 ± 1.10	3.00 ± 1.50	0.07

CD-RISC	29.07 ± 6.16	29.87 ± 5.86	0.59	30.02 ± 6.56	28.51 ± 5.51	0.04

Values are age-corrected *t*-scores (mean ± SD, 50 ± 10) for the POMS subscales and raw score mean ± SD for the CD-RISC and the Brief COPE.

a Probability associated with an independent samples *t*-test (two-tailed distribution).

b Percentage of participants impaired on the POMS Tension/Anxiety and Depression scales.

c Probability associated with a chi-square test (two-tailed distribution).

**Table 3:** Perceived environmental and health risks of oil spill.

	Exposure			Income status		
Perceptions	Indirect (*n* = 71)	Direct (*n* = 23)	*p*-Value^a^	Stable (*n* = 47)	Loss (*n* = 47)	*p*-Value^a^
Worry about environment	68 (99)	23 (100)	0.56	98	100	0.32
Worry about seafood safety	68 (99)	23 (100)	0.56	98	100	0.32
Believe BP will succeed in cleanup	27 (42)	3 (15)	0.03	41	31	0.36
Science will succeed in cleanup	35 (55)	8 (40)	0.25	49	54	0.67
Worry about human health	66 (96)	22 (96)	1.00	96	96	1.00
Duration of health effects	NA	NA	0.03	NA	NA	0.52
Short term	11 (21)	0 (0)	NA	7 (18)	4 (12)	NA
Long term	42 (79)	20 (100)	NA	33 (83)	29 (88)	NA
Believe they had exposure-related illness	0 (0)	2 (9)	0.04	1 (2)	1 (2)	0.45
Don’t know if exposure-related illness	11 (17)	5 (22)		6 (13)	10 (23)	

NA, not applicable. Responses for each exposure group are reported as frequencies (percentages).

a Probability associated with a chi-square test (two-tailed distribution)
